# Targeting of PP2 A/GSK3β/PTEN Axis in Alzheimer Disease: The Mooting Evidence, Divine, and Devil

**DOI:** 10.1007/s10571-025-01554-0

**Published:** 2025-04-18

**Authors:** Saad Misfer Alqahtani, Hayder M. Al-Kuraishy, Ali I. Al-Gareeb, Maha M. Abdel-Fattah, Ahad Amer Alsaiari, Mubarak Alruwaili, Marios Papadakis, Athanasios Alexiou, Gaber El-Saber Batiha

**Affiliations:** 1 Department of Pathology, College of Medicine, The University Hospital, Najran University, Najran, Saudi Arabia; 2 Department of Clinical Pharmacology and Medicine, College of Medicine, Mustansiriyah University, Baghdad, Iraq; 3 Department of Clinical Pharmacology, Jabir Ibn Hayyan Medical University, Al-Ameer Qu./Najaf-Iraq, Po. Box (13), Kufa, Iraq; 4 Department of Pharmacology and Toxicology, Faculty of Pharmacy, Beni-Suef University, Beni-Suef, 62514 Egypt; 5 Department of Clinical Laboratory Science, College of Applied Medical Science, Taif University, Taif, Saudi Arabia; 6 Department of Internal Medicine, College of Medicine, Jouf University, Sakaka, Saudi Arabia; 7 University Hospital Witten-Herdecke, University of Witten-Herdecke, Heusnerstrasse 40, 42283 Wuppertal, Germany; 8 University Centre for Research & Development, Chandigarh University, Mohali, India; 9 Department of Science and Engineering, Novel Global Community Educational Foundation, Hebersham, NSW Australia; 10 Department of Research & Development, Funogen, Athens, Greece; 11 Department of Pharmacology and Therapeutics, Faculty of Veterinary Medicine, Damanhour University, Damanhour, 22511 AlBeheira Egypt

**Keywords:** Alzheimer disease, Phosphatase 2 A (PP2 A), Glycogen synthase kinase 3β (GSK3β), Phosphatase and tensin homologue (PTEN)

## Abstract

Alzheimer disease (AD) is a progressive neurodegenerative disease of the brain due to extracellular accumulation of Aβ. In addition, intracellular accumulation of hyperphosphorlyated tau protein which form neurofibrillary tangle (NFT) is associated with progressive neuronal injury and the development of AD. Aβ and NFTs interact together to induce inflammation and oxidative stress which further induce neurodegeneration in AD. The exact relationship between Aβ and tau, the two proteins that accumulate within these lesions, has proven elusive. A growing body of work supports the notion that Aβ may directly or indirectly interact with tau to accelerate NFTs formation. Aβ can adversely affect distinct molecular and cellular pathways, thereby facilitating tau phosphorylation, aggregation, mislocalization, and accumulation. Aβ may drive tau pathology by activating specific kinases, providing a straightforward mechanism by which Aβ may enhance tau hyperphosphorylation and NFT formation. Many cellular signaling pathways such as protein phosphatase 2A (PP2A), glycogen synthase kinase 3β (GSK3β), and phosphatase and tensin homologue (PTEN) are intricate in AD neuropathology. PP2A which involved in the dephosphorylation of tau protein is deregulated in AD, and correlated with cognitive impairment. PTEN is a critical regulator of neuronal growth, survival, and development, improving synaptic plasticity and axonal regeneration. Nevertheless, mutated PTEN is associated with the development of cognitive impairment by inhibiting the expression and the activity of PP2A. Furthermore, dysregulation of GSK3β affects Aβ, tau protein phosphorylation, synaptic plasticity and other signaling pathways involved in the pathogenesis of AD. Therefore, there is a close interaction among GSK3β, PTEN, and PP2A. GSK3β exaggerates AD neuropathology by inhibiting PP2A and activates the expression of PTEN. These findings specified a related interaction among GSK3β, PTEN, and PP2A, and modulation of the single component of this axis may not produce an effective effect against AD neuropathology. Modulation of this axis by metformin and statins can reduce AD neuropathology. Therefore, this review aims to discuss the role of GSK3β/PTEN/PP2A axis in AD neuropathology and how targeting of this axis by metformin and statins can produce effective therapeutic strategy in the management of AD. In conclusion, inhibition of GSK3β and PTEN and activation of PP2A may be more suitable than modulation of single signaling pathway. Metformin and statins by activating PP2A and inhibiting of GSK3β and PTEN attenuate the development and progression of AD.

**Graphical Abstract:**

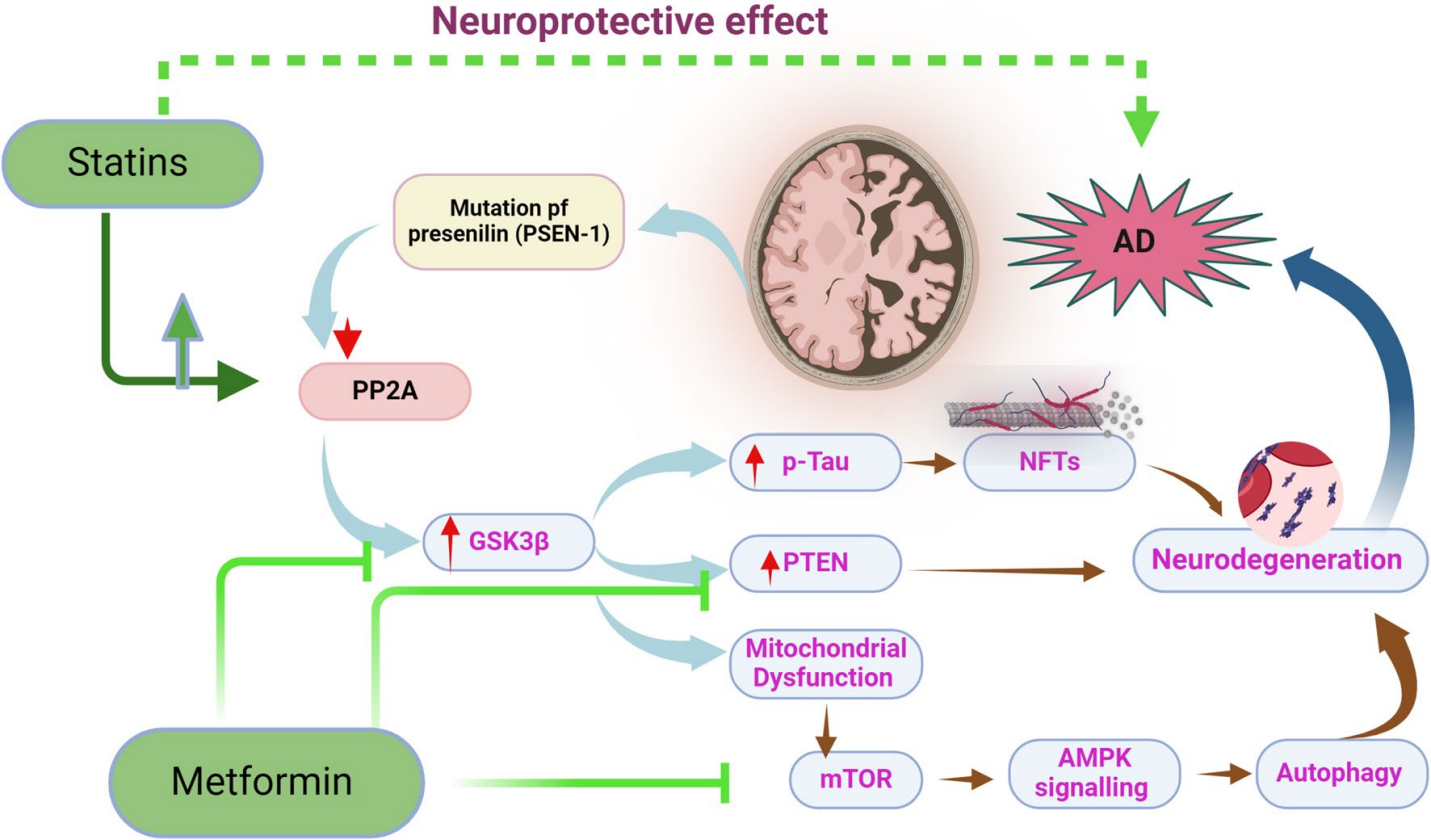

## Introduction

**Fig. 1:**
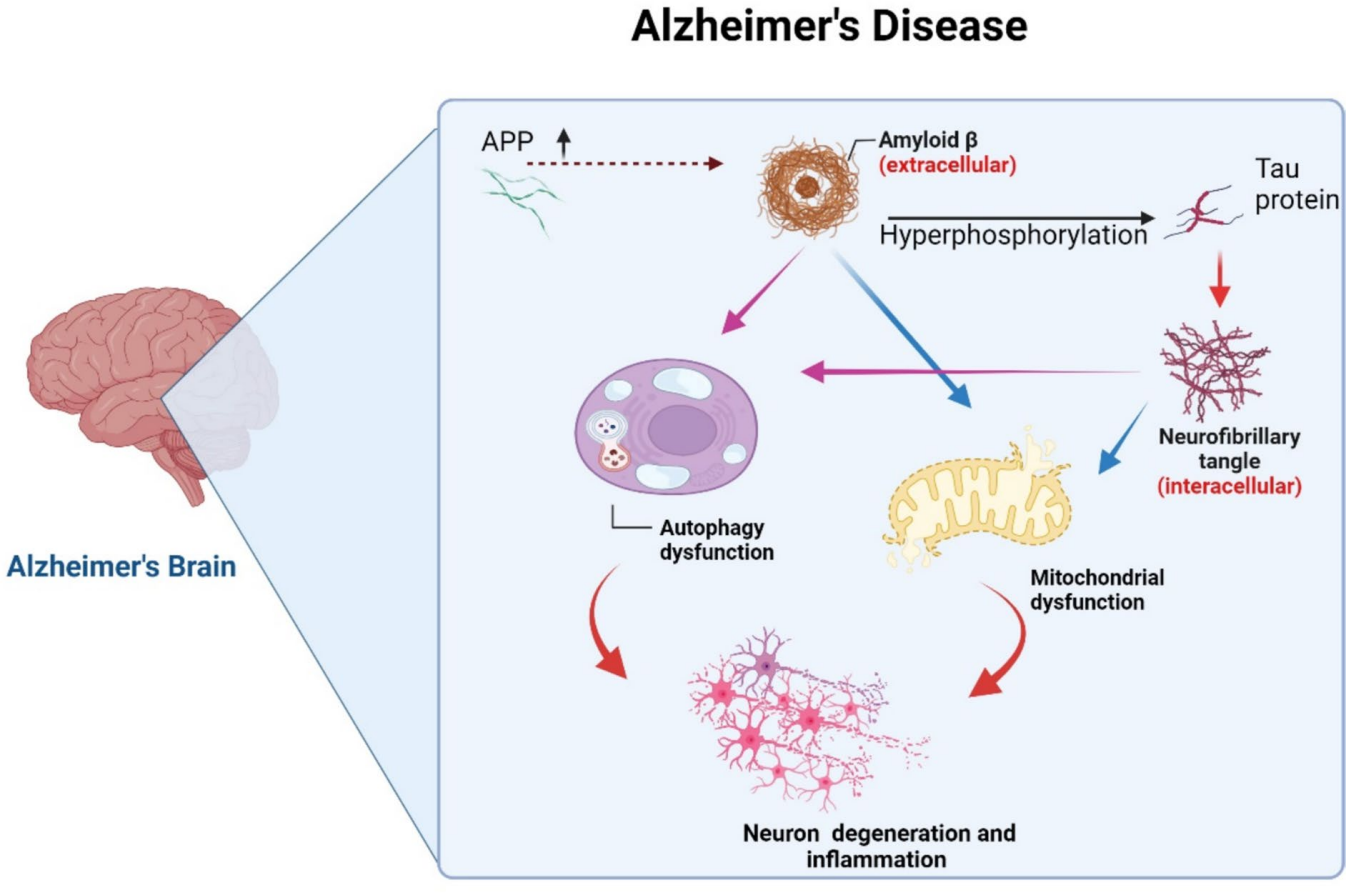
The pathophysiology of AD: Overproduction of Aβ from mutant amyloid precursor protein (APP) or defect in the clearance of Aβ increase the accumulation of Aβ which induce the tau protein hyperphosphorylation and the formation of neurofibrillary tangle (NFT). These neuropathological changes trigger the development of mitochondrial dysfunction, neuronal apoptosis, autophagy dysfunction and progressive neurodegeneration, and the development of AD

Alzheimer disease (AD) is the most common neurodegenerative disorder and the sixth most common cause of death in the USA. AD is the most common cause of dementia represents 75% of all dementia types (Rawat et al. 2022). Of note, there are two types of AD, sporadic AD forms 90% of AD and familial AD forms 10% of AD. Sporadic AD is more correlated with old age > 65 year and late-onset AD, although familial AD is linked with the development of early-onset AD (Scheltens et al. 2021; Rujeedawa et al. 2021). AD is a progressive neurodegenerative disease of the brain due to extra-neuronal accumulation of Amyloid beta (Aβ) protein in the brain with subsequent neuronal apoptosis (Al-Kuraishy et al. 2023d). Increasing accumulation of Aβ is due to either overproduction of Aβ from mutant amyloid precursor protein (APP) or defect in the clearance of Aβ (Scheltens et al. 2021; Trejo-Lopez et al. 2023; Porsteinsson et al. 2021). In addition, intracellular accumulation of hyperphosphorlyated tau protein which form neurofibrillary tangle (NFT) is associated with progressive neuronal injury and the development of AD (Ju and Tam 2022). Aβ and NFTs interacts together to induce inflammation and oxidative stress which further induce neurodegeneration in AD (Rujeedawa et al. 2021). The exact relationship between Aβ and tau, the two proteins that accumulate within these lesions, has proven elusive. A growing body of work supports the notion that Aβ in AD and old age may directly or indirectly interact with tau to accelerate NFTs formation. Aβ can adversely affect distinct molecular and cellular pathways, thereby facilitating tau phosphorylation, aggregation, mislocalization, and accumulation (Kumar and Bansal 2022; Sequeira and Godad 2024). The putative mechanisms by which Aβ may facilitate the development of tau pathology are numerous. A great deal of work suggests that Aβ may drive tau pathology by activating specific kinases, providing a straightforward mechanism by which Aβ may enhance tau hyperphosphorylation and NFT formation. In the AD brain, Aβ also triggers a massive inflammatory response and pro-inflammatory cytokines can in turn indirectly modulate tau phosphorylation. Mounting evidence also suggests that Aβ may inhibit tau degradation via the proteasome (Kumar and Bansal 2022; Sequeira and Godad 2024). Aβ and tau may indirectly interact at the level of axonal transport and evidence is presented for two possible scenarios by which axonal transport deficits may play a role [151–156]. Therefore, AD neuropathology is complex and related to different cellular and sub-cellular disorders (Fig. 1).

**Fig. 2:**
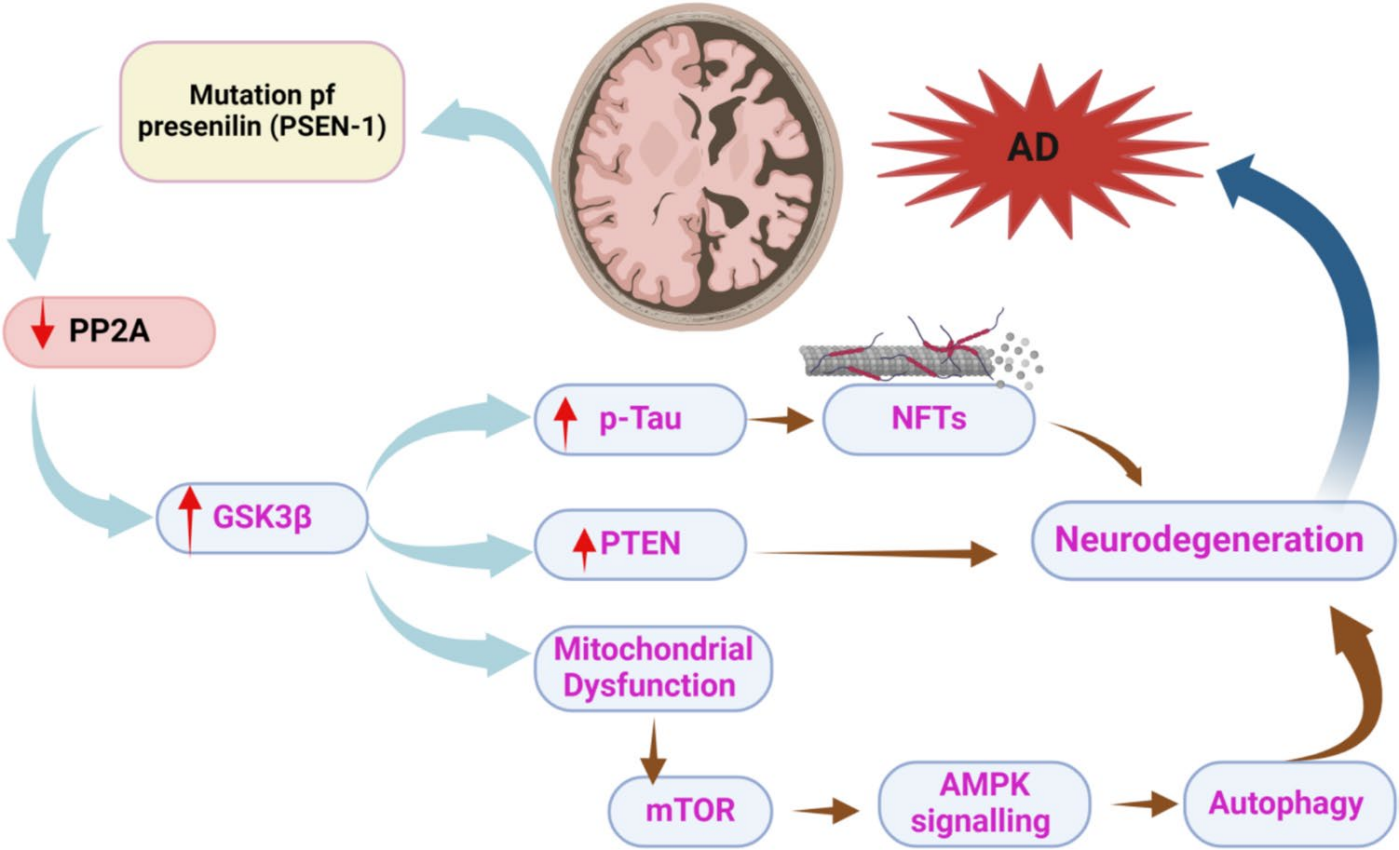
Dysregulation of signaling pathways in AD: Mutation of *PSEN-1* gene triggers the activation of GSK3β signaling pathway through inhibition of the neuroprotective PP2 A. Augmentation of GSK3β signaling pathway leads to mitochondrial dysfunction and activation of mTOR signaling, which through inhibition of AMPK inhibits autophagy and induces neurodegeneration. GSK3β by activating PTEN results in direct neurodegeneration. In addition, over-activated GSK3β promotes tau protein hyperphosphorylation with formation of NFTs which induce neurodegeneration. Progressive neurodegeneration is linked with the development and progression of AD

It has been shown that different cellular signaling pathways, such as protein phosphatase 2 A (PP2 A), glycogen synthase kinase 3 β (GSK3β), and phosphatase and tensin homologue (PTEN) are involved in AD neuropathology (Kumar and Bansal 2022; Sequeira and Godad 2024). PP2 A which involved in the dephosphorylation of tau protein is deregulated in AD and correlated with cognitive impairment in mouse model (Leong et al. 2020). PTEN is a critical regulator of neuronal growth, survival and development, improving synaptic plasticity and axonal regeneration (Shen et al. 2021). However, mutated and overexpressed PTEN is linked with the development of cognitive impairment in mouse model by inhibiting the expression and the activity of PP2 A (Shen et al. 2021). In addition, dysregulation of GSK3β affects Aβ, tau protein phosphorylation, synaptic plasticity, and other signaling pathways are involved in the pathogenesis of AD (Lauretti et al. 2020). Findings from preclinical studies demonstrated a close interaction among GSK3β, PTEN, and PP2 A (Liu et al. 2008; Mai et al. 2021). For example, GSK3β exaggerates AD neuropathology by inhibiting PP2 A (Liu et al. 2008). Moreover, GSK3β activates the expression of PTEN (Mai et al. 2021). These findings indicated a mutual interaction among GSK3β, PTEN, and PP2 A (Fig. 2), and modulation a single component of this axis may not produce an effective effect against AD neuropathology. Therefore, this review tries to discuss targeting of this axis in AD and how FDA-approved drugs affect this axis and produce an effective therapeutic strategy in the management of AD.

## Role of PP2 A/PTEN/GSK3β Axis in AD

### PP2 A and AD

**Fig. 3:**
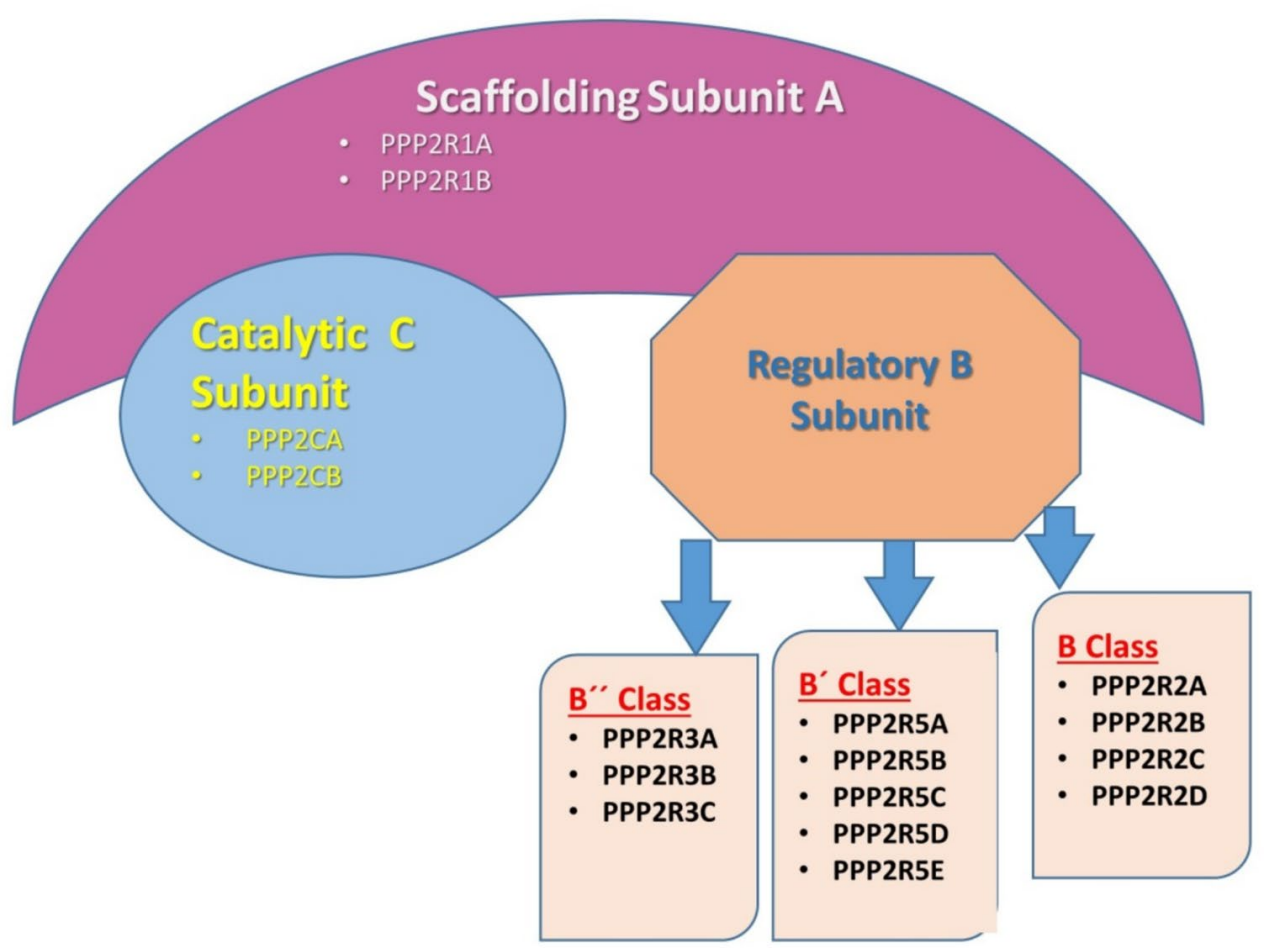
Regulatory and catalytic subunits of PP2 A: PP2 A is composed of three components: the structural A subunit, the regulatory B subunit, and the catalytic C subunit with distinct characteristics and functions

PP2 A is a ubiquitous, highly conserved family of at least 96 serine/threonine phosphatases that represent 0.1–1% of total cellular proteins and play a crucial role in regulating most cellular functions. PP2 A is a large family of enzymes and account for the most of brain phosphatase activity. PP2 A composed of three components: the structural A subunit, the regulatory B subunit, and the catalytic C subunit with distinct characteristics and functions (Sandal et al. 2021). PP2 A regulation is highly complex, involving not only the interplay of specific regulatory subunits and modulators but also post-translational modifications, protein–protein interactions, and sub-cellular compartmentalization (Sandal et al. 2021) (Fig. 3).

PP2 A has a neuroprotective effect against AD development by inhibiting amyloidogenesis and tau protein hyperphosphorylation (Sontag and Sontag 2014). However, alteration of the catalytic activity and regulators of PP2 A and reduction of PP2 A are implicated in the pathogenesis of AD by increasing of amyloidogenesis and tau protein hyperphosphorylation and impairment of synaptic plasticity (Sontag and Sontag 2014). Deregulation of PP2 A enzymes also affects the activity of many protein kinases implicated in AD. The PP2 A/Bα isoform binds to tau and is the primary tau phosphatase. Its deregulation correlates with increased tau phosphorylation in vivo and in AD. Disruption of PP2 A/Bα-tau protein interactions likely contribute to tau deregulation in AD deregulation of PP2 A methylation with down-regulation of PP2 A/Bα, enhanced phosphorylation of tau and amyloid precursor protein, tau mislocalization, microtubule destabilization and neuritic defects (Sontag and Sontag 2014). It has been shown that total activity of PP2 A was decrease in AD cortical and hippocampal brain homogenates (Theendakara et al. 2017). As well, experimental studies also point to an association between PP2 A deregulation and amyloidogenesis, but underlying mechanisms remain unclear (Taleski et al. 2021). Reduced PP2 A methylation promotes the accumulation of both phosphorylated tau and APP isoforms and increased secretion of β-secretase-cleaved APP fragments and Aβ. Conversely, incubation of N2a cells with S-adenosylmethionine and expression of PPMT enhance PP2 A methylation. This leads to the accumulation of dephosphorylated tau and APP species and increased secretion of neuroprotective α-secretase-cleaved APP fragments (Taleski et al. 2021). Numerous abnormalities of PP2 A have been reported in AD, including among others decreased protein levels of PP2 A, decreased mRNA and protein levels of the catalytic subunit PP2 A_C_ and variable regulatory B subunits and reduced methylation of the catalytic subunit, all of which results in disruption of the PP2 A phosphatase activity. ApoE transcriptionally represses PPP2R5E and reduces protein expression, ApoE triggers demethylation of the catalytic subunit of PP2 A. Down-regulation of PPP2R5E gene expression and reduction in PP2 A activity by ApoE4 compared with ApoE3 (Theendakara et al. 2017). This may also explain an elevated tau phosphorylation in AD human brains that featured at least one ApoE4 allele. Thus, ApoE reduces the PP2 A catalytic activity that has implications for AD. Deficits in PP2 A activity are harmonized with the reported down-regulation of PP2 A catalytic C subunit at the gene, mRNA, and protein expression levels in AD (Shentu et al. 2018). However, PP2 A expression levels are increased in AD astrocytes (Fan et al. 2022). Findings from preclinical study demonstrated that astrocytic lactoferrin enhances PP2 A activity and inhibits Aβ production via APP dephosphorylation in APP/PS1 mice (Fan et al. 2022). Therefore, promoting astrocytic lactoferrin expression may be a potential strategy against AD. More specifically, decreased expression levels of PP2 A regulatory subunit mRNAs in the hippocampus and cortical Bα subunit have been reported in AD (Sontag and Sontag 2014). Particularly, the loss of neuronal PP2 A/Bα holoenzymes correlates with the down-regulation of PP2 A methylation and severity of phosphorylated tau pathology in AD-affected brain regions (Sontag et al. 2014). Considerably, down-regulation of LCMT1 protein expression matches the deficits in PP2 A methylation observed in AD (Sontag et al. 2014). Up-regulation of I1PP2 A and I2PP2 A, and mislocalization and cleavage of I2PP2 A, could trigger the inactivation of PP2 A in AD neocortical neurons (Yang 2023). Reduced expression levels of PTPA in AD brain tissue may also lead to inactivation of PP2 A indirectly by increasing levels of PP2 A phosphorylated at the Tyr-307 site (Javadpour et al. 2019). PP2 A dysfunction can promote aberrant stimulation of signaling cascades that contribute to neuronal and synaptic damage in AD. Contrariwise, some Ser/Thr protein kinases can in turn modulate PP2 A. For example, activated GSK3β induces PP2 A inactivation via several mechanisms: phosphorylation of PP2 A on Tyr-307, demethylation of PP2 A on Leu309 through inhibition of LCMT1 and up-regulation of PME1, and accumulation of I2PP2 A (Lin et al. 2007). Besides Ser/Thr kinases, the protein tyrosine kinase promotes the phosphorylation of PP2 A on Tyr-307, resulting in PP2 A inactivation and subsequent tau phosphorylation (Lin et al. 2007). Nevertheless, the interconnection between the deregulation of these various protein kinase/PP2 A-dependent signaling cascades and AD pathogenesis is quiet indistinct. Therefore, PP2 A dysfunction is a key player in the development of tau pathology in AD.

**Fig. 4:**
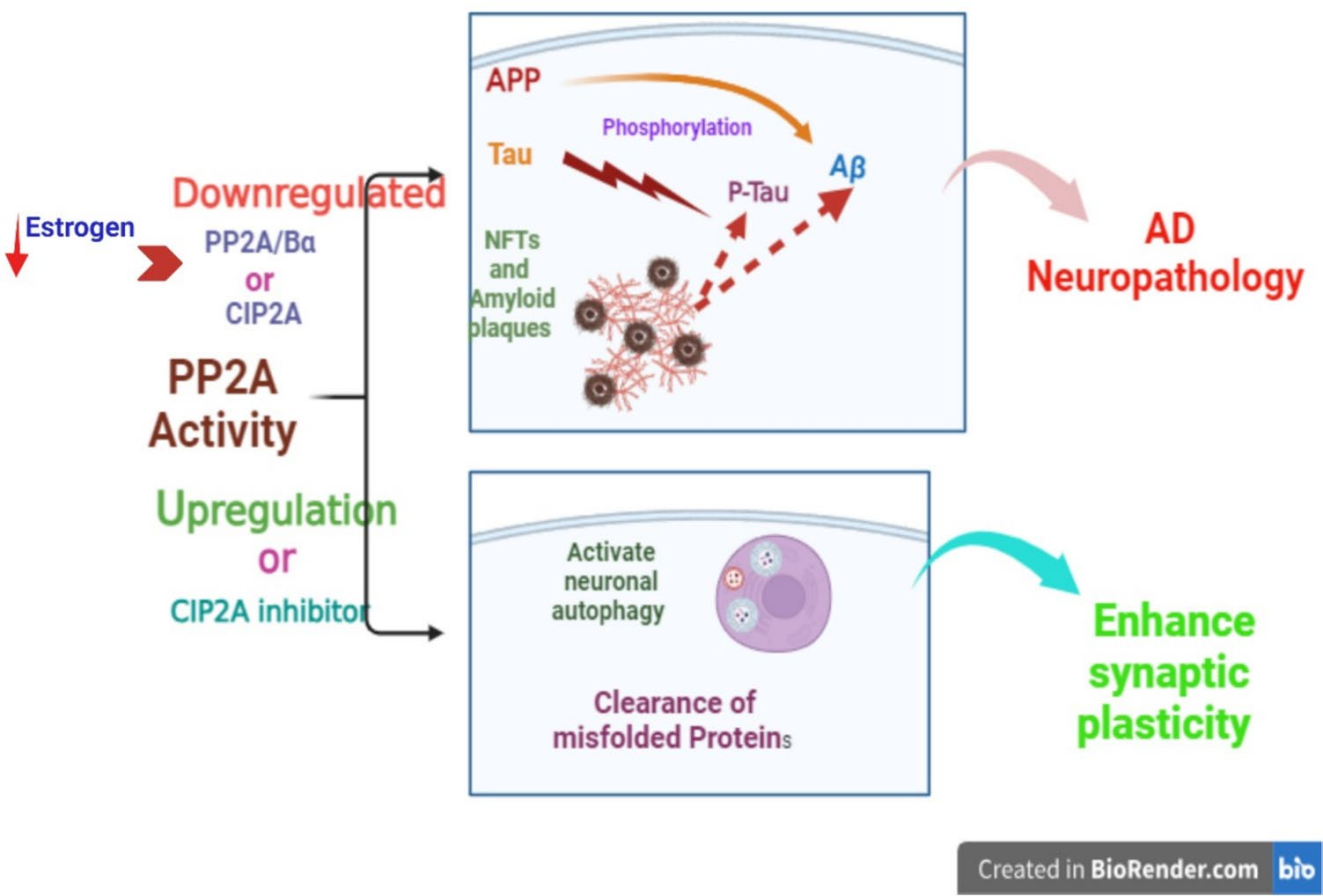
The neuroprotective effect of PP2 A: PP2 A is negatively regulated by cancerous inhibitor of PP2 A (CIP2 A). Therefore, activation of PP2 A and inhibition of CIP2 A prevent APP processing for the generation of neurotoxic Aβ and reduce the formation of NFTs therefore attenuate neurodegeneration and AD neuropathology. In addition, PP2 A activates neuronal neuropathology and enhances the clearance of misfolded proteins thereby improving synaptic plasticity in AD

PP2 A enzymes also oppose the activity of many brain protein kinases up-regulated in AD (Lin et al. 2007; Taleski et al. 2021). PP2 A deregulation affects the activity of different serine/threonine enzymes which implicated in the pathogenesis of AD. PP2 A/Bα binds and induces dephosphorylation of tau protein, and down-regulation of PP2 A/Bα is associated tau protein hyperphosphorylation and the development of AD (Sontag and Sontag 2014; Sandal et al. 2021). In addition, impairment of PP2 A is linked with the development of sporadic AD due to mislocalization of tau protein and enhancement the phosphorylation of APP (Zhou et al. 2020). Moreover, up-regulation of PP2 A inhibitor known as cancerous inhibitor of PP2 A (CIP2 A) is associated with AD neuropathology. Inhibition of the CIP2 A by the natural product polyphyllin can attenuate AD-like pathology in 3xTg mice by reactivating of brain PP2 A, and enhancement of synaptic plasticity (Zhou et al. 2020). In addition, cell cycle checkpoint protein kinase 1 (Chk1) which is a serine/threonine kinase and inhibit PP2 A, is activated in response to the DNA damage in AD. Activated Chk1 triggers the expression of CIP2 A which inhibit the activity of PP2 A in AD cell model and in transgenic APP mice (Hu et al. 2022). Consistently, PP2 A is downregulated in AD mouse model (Leong et al. 2020). Moreover, mutation of ApoE4 which intricate in the pathogenesis of AD inhibits the expression and activity of PP2 A in human brains of AD patients (Theendakara et al. 2017). Of note, PP2 A activates neuronal autophagy and enhances the clearance of misfolded proteins. Suppression of PP2 A leads to the inhibition of autophagy activity and exaggeration of AD neuropathology (Magnaudeix et al. 2013). Therefore, down-regulation of PP2 A is secondary to AD as Aβ and hyperphosphorylated tau protein also inhibit the expression and activity of PP2 A (Arnaud et al. 2011). Furthermore, hyperhomocysteinemia increase risk of AD by inhibiting the expression of PP2 A in rat hippocampus (Zhang et al. 2008). Moreover, the neuroprotective effect of estrogen hormone is mediated by upregulating PP2 A expression and activity (Don Yi and Simpkins 2008). Thus, hyperhomocysteinemia and estrogen deficiency increases risk of AD neuropathology by inhibiting the expression of PP2 A (Fig. 4). These findings indicated that PP2 A is deregulated in AD due to activation of PP2 A inhibitors. Therefore, restoration of PP2 A expression and activity can reduce the pathogenesis of AD.

Therefore, there is growing interest in developing PP2 A-targeted therapies for AD, especially to counteract tau pathology. Many compounds modulate PP2 A activity by numerous direct and indirect mechanisms. It has been shown that the activity of PP2 A can be regulated by direct allosteric activators or suppression of PP2 A inhibitors (Voronkov et al. 2011). In vivo studies have shown that some experimental compounds and drugs currently used clinically can activate PP2 A and consequently reverse AD-like tau phosphorylation and associated cognitive impairment, for instance, sodium selenate, by increasing PP2 A activity through unknown mechanisms. Sodium selenite increases the activity of PP2 A in neuroblastoma cell line and in aged mice (Corcoran et al. 2010). Sodium selenite improves the cognitive function in transgenic mice by reducing tau protein level in the amygdala and hippocampus in transgenic mice by upregulating of PP2 A activity (Corcoran et al. 2010). However, this effect is reversible, thus prolong use of sodium selenite is recommended (Corcoran et al. 2010). In addition, synthetic tricyclic sulfonamide reduced AD neuropathology in AD cell model and rat model by activating the expression of PP2 A (Wei et al. 2020). As ell, Asle-Rousta et al. (Asle-Rousta et al. 2013) found that fingolimod attenuated cognitive impairment in AD rat model by increasing the activity of PP2 A. Similarly, ApoE mimetic peptide COG1410 improves cognitive impairment in transgenic AD mouse model by activating the activity of PP2 A (Vitek et al. 2012). Similarly, a non-steroidal anti-inflammatory drug tolfenamic acid attenuates AD neuropathology in animal model by increasing the activity of PP2 A (Wang et al. 2021b). Furthermore, the anti-AD drug memantine prevents the I2PP2 A-induced inhibition of PP2 A activity in vitro. These findings demonstrate novel mechanisms by which I2PP2 A regulates the intracellular activity of PP2 A and phosphorylation of tau, and by which memantine modulates PP2 A signaling and inhibits NFT-induced neurodegeneration (Chohan et al. 2006).

These findings highlighted that activation of PP2 A could be a therapeutic strategy in the management of AD. It is improbable that a single compound will overcome all the many-sided PP2 A deficits that are present in AD. It is not clear either how exactly pharmacological PP2 A activation will restore the collective function of specific PP2 A holoenzymes and PP2 A modulators that become down-regulated at the protein level in AD neurons. Drugs may also target PP2 A enzymes outside the brain and in brain regions not primarily involved in the disease process (Lambrecht et al. 2013). Furthermore, pathological brain changes manifest at least one decade before appearance of AD symptoms (Tahami Monfared et al. 2022). Thus, one need to take into account that treatment with pharmaceutical PP2 A drugs may require to be started early and prolonged for a long period of time, which increases chances for compensatory mechanisms and side effects. More meaningfully, they do not exhibit the metabolic deficits observed in AD patients. For instance, epidemiological studies show that AD patients have low plasma folate status which can negatively impact PP2 A methylation (Zhang et al. 2021a). Therapeutic approaches may also need to take into account the existence of common human polymorphisms and age-related epigenetic changes in folate-related genes that affect folate status and have been linked with late-onset AD (Zhang et al. 2021a). The resulting impairment of methylation pathways may offset the efficacy of potential PP2 A agonists.

### PTEN and AD

PTEN is an essential phosphatase protein found in both nucleus and cytoplasm, involved in the regulation of cell proliferation, growth, adhesion, apoptosis, and migration (Wang et al. 2021b; Huang et al. 2021). The importance of PTEN in cellular function is underscored by the frequency of its deregulation in cancer. PTEN tumor-suppressor activity depends largely on its lipid phosphatase activity, which opposes the neuroprotective PI3 K/AKT activation. It has been illustrated that the phenylpropanoid glycoside salidroside isolated from *Rhodiola rosea L*., protects against Aβ-induced neurotoxicity in Drosophila AD models by upregulating of PI3 K/Akt signaling (Zhang et al. 2016).

As such, PTEN regulates many cellular processes, including proliferation, survival, energy metabolism, cellular architecture, and motility. More than a decade of research has expanded our knowledge about how PTEN is controlled at the transcriptional level as well as by numerous posttranscriptional modifications that regulate its enzymatic activity, protein stability, and cellular location (Wang et al. 2021b; Huang et al. 2021). Although the role of PTEN in cancers has long been appreciated, it is also emerging as an important factor in other diseases, such as diabetes and autism spectrum disorders (Wang et al. 2021b; Huang et al. 2021).It maintains the stability of DNA and chromosome genome through modulation transcription and translation of proteins by regulating PI3 K and AKT signaling. PI3 K/AKT which inhibited by PTEN promotes cell proliferation and tumor growth (Huang et al. 2021; Wang et al. 2021b). Therefore, PTEN is regarded as a tumor suppressor protein by inhibiting the excessive cellular growth. It controls the axonal growth in the CNS by regulating the differentiation of neural stem cells (Shen et al. 2020). Interfering with the expression of PTEN promotes neurite outgrowth in ischemic stroke model (Shen et al. 2020). Deletion of *PTEN* gene promotes synaptic plasticity and hippocampal excitatory synapses (Fraser et al. 2008). Sonoda et al. (2010) illustrated that PTEN expression was altered in the postmortem brains of AD patients compared to controls. PTEN negatively regulates intracellular levels of PIP3 and antagonizes the PI3 K signaling pathway important for cell survival. PTEN is mislocalized and accumulated in the NFTs of AD patients leading to the inhibition of PI3 K and PP2 A. Intense immunolabeling was found in the large neurons, such as pyramidal cells, although reduced expression and redistribution of PTEN in the remaining neurons in AD. PTEN is redistributed in damaged neurons from the nucleus and cytoplasm to neuritic pathology such as NFTs, neuropil threads, and dystrophic neurites within senile plaques in AD hippocampus, subiculum, entorhinal cortex, and angular gyrus. Also, double immunofluorescence staining showed dual labeling of intracellular NFTs for PTEN and tau, labeling of some axons for PTEN and phosphorylated neurofilament, and weak labeling of a few reactive astrocytes around senile plaques for PTEN. Double labeling of NFTs is observed in a subset of tangle-bearing neurons either for PTEN and GSK3β or for PTEN and MEK (Sonoda et al. 2010). Therefore, delocalized PTEN causes a deregulation of PI3 K pathway in the cytoplasm and may induce the nuclear dysfunction of PTEN in AD degenerating neurons. As well, PTEN is implicated in the development of excitotoxicity and mitochondrial apoptosis in transgenic mice (Grande et al. 2014). Excitotoxic damage exemplifies the major mechanism leading to cell death in many human neurodegenerative diseases including AD that caused by a glutamate excess and associated excitotoxicity independent of PP2 A inhibition. PTEN is considered as a downstream the excitatory receptors activated in excitotoxicity involved in neuronal damage and induction of reactive astrogliosis (Grande et al. 2014). Inhibition of PTEN rescues neuronal death and decreases the reactive astrogliosis in the hippocampus caused by kainite which increases mitochondrial PTEN (Grande et al. 2014). Of interest, APP promotes the expression of PTEN result in the inhibition of PP2 A and the accumulation of tau protein which cause axonal degeneration and synaptic dysfunction in AD model (Goiran et al. 2018). Increased the levels of PP2 A phosphorylated in tyrosine 307 in the hippocampus and the cerebellum and significantly decreased the levels of PTEN phosphorylated in serine 380 in the hypothalamus and in the hippocampus through PI3 K/Akt and ERK1/2 (Goiran et al. 2018). PTEN inhibits the functional activity of PI3 K/Akt and ERK1/2 leading to cognitive impairment in animal models (Goiran et al. 2018).

APP intracellular domain (AICD) accounts for PSEN-dependent phenotype and upregulates PTEN transactivation in cells as well as *in vivo* in a Forkhead box O3a-(FOXO3a)-dependent manner (Grande et al. 2014). The modulation of γ-secretase activity or AICD expression affects PTEN-related control of mitophagy and mitochondrial dynamics (Zou et al. 2020).

Moreover, up-regulation of GSK3β in AD promotes the expression of PTEN (Al-Khouri et al. 2005). PTEN is regulated by phosphorylation on multiple serine and threonine residues in its C terminus by casein kinase 2 (CK2). However, CK2 does not phosphorylate all sites in PTEN and that GSK3β also contributes in PTEN phosphorylation. Although CK2 mainly phosphorylated PTEN at Ser-370 and Ser-385, GSK3β phosphorylated Ser-362 and Thr-366. Thus, PTEN phosphorylation is mediated GSK3β (Al-Khouri et al. 2005). Findings from preclinical study observed that hyperphosphorylated tau protein activates the expression of PTEN which induce neuronal loss and synaptic injury through activation of microglia in AD mouse model (Benetatos et al. 2020). Pathological tau protein encourages early activation of PTEN, which precedes apoptotic caspase-3 cleavage in transgenic mouse model. Pharmacological inhibition of PTEN reduces uptake of tau protein by microglia uptake in transgenic mice (Benetatos et al. 2020). Therefore, mutant and over-activated PTEN is intricate in the pathogenesis of AD by inducing synaptotoxicity and tauopathy. Thus, inhibition of PTEN may reduce AD neuropathology through PP2 A-mediated synaptic plasticity and inhibition of tauopathy.

It has been observed that PTEN inhibitor bisperoxovanadium has a neuroprotective effect in many neurological disorders (Mao et al. 2015). Moreover, bisperoxovanadium treatment meaningfully reduced PTEN mRNA and protein levels and increased PI3 K, Akt, and GSK-3β proteins expression in the ischemic boundary zone of the cerebral cortex at 4 days after middle cerebral artery occlusion (Mao et al. 2015). Therefore, bisperoxovanadium may be effective against AD neuropathology (He et al. 2021). Bisperoxovanadium inhibits neuro-inflammation in both Aβ-stimulated BV2 microglial cell line and APP transgenic mouse brain. Bisperoxovanadium administration considerably decreased the levels TNF-α, IL-6, IL-1β, and cyclooxygenase-2 both in the hippocampus of APP mice and in the Aβ-stimulated BV2 microglia. Furthermore, bisperoxovanadium inhibits the Aβ-induced activation of NF-κB signaling and up-regulated the protein expression level of peroxisome proliferator-activated receptor gamma (PPARγ) in a dose-dependent manner. Consistently, PPARγ inhibitor GW9662 eliminates the effect of bisperoxovanadium on Aβ-induced NF-κB activation and pro-inflammatory mediator production (He et al. 2021). In addition, a plant sarcopoterium inhibits the expression and activity of PTEN thereby reducing the development and progression of AD (Rozenberg et al. 2014; Ben-Shachar et al. 2019). Sarcopoterium spinosum inhibited PTEN and activated PKB by a mechanism which is independent of ser473 and thr308 phosphorylation. Other post-translation modifications might be involved and should be analyzed further in order to understand this unique PKB activation. Identifying the active molecules in the extract, may lead to the development of new agents for the treatment of insulin resistance (Rozenberg et al. 2014; Ben-Shachar et al. 2019).

**Fig. 5:**
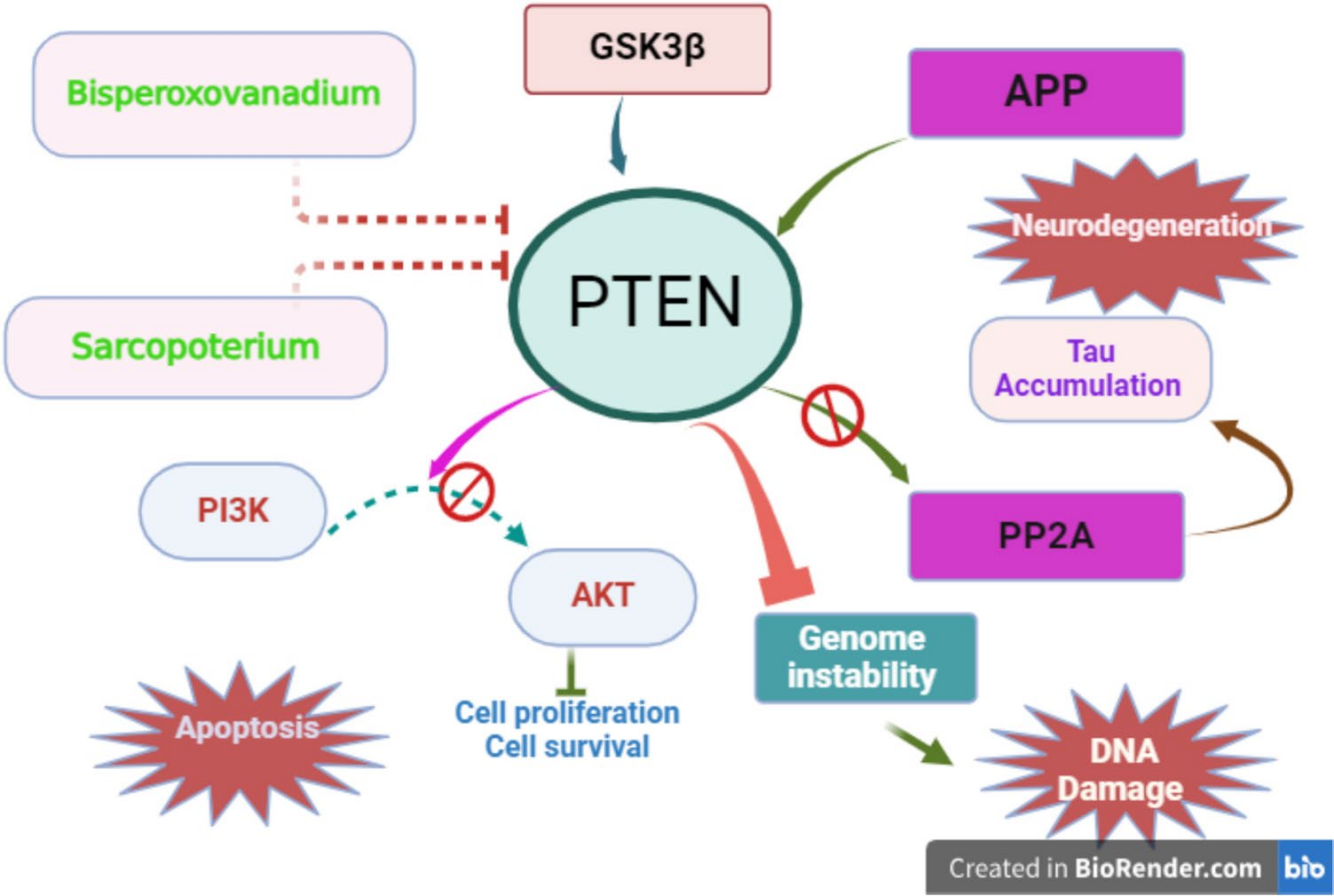
Role of PTEN in AD: PTEN is activated by mutant GSK3β and APP leading to inhibition of neuronal proliferation, induction of DNA injury, and neuronal apoptosis through PI3 K/AKT signaling pathway. PTEN inhibitors attenuate AD neuropathology by inhibiting PTEN-mediated DNA damage and neuronal apoptosis

These findings highlighted that overexpression of PTEN is linked with AD neuropathology, therefore inhibition of this phosphates can attenuate the pathogenesis of AD (Fig. 5).

### GSK3β and AD

**Fig. 6:**
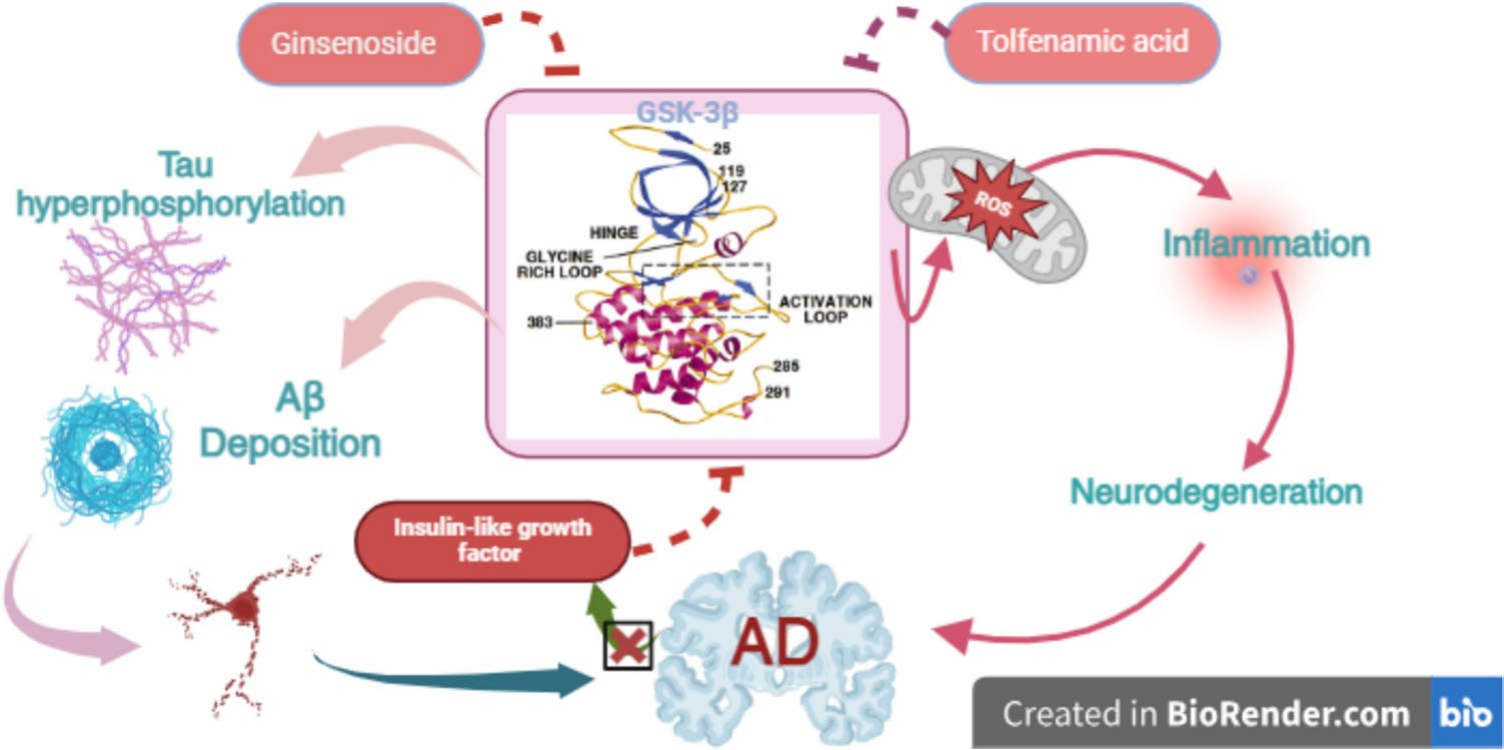
Role of GSK3β in AD: GSK3β triggers AD neuropathology by inducing the accumulation of tau protein and Aβ. In addition, GSK3β induces inflammation and neurodegeneration by inducing the generation of ROS in AD. GSK3β inhibitors including ginsenoside and tolfenamic acid reduce AD neuropathology

GSK3β is a conserved serine/threonine protein kinase involved in the phosphorylation of glycogen synthase. It is highly expressed in the all brain areas and intricate in the regulation of many signaling pathways (Turkistani et al. 2024). Biochemical signaling pathways are known to have a critical role in neuronal development and function. A growing body of evidence is accumulating to suggest that signaling pathways also trigger neurodegeneration and neurodegenerative disease. One pathway with a prominent role in neurodegenerative disease is the signaling pathway in which the GSK3β is a key component. In vitro and in vivo evidences point to a key role for GSK3β in promoting neurodegeneration and in AD plaque and NFTs formation (Turkistani et al. 2024). How GSK3β acts in this regard is still open to debate, but it may involve both extracellular and nuclear apoptotic activities. GSK3β regulates the expression of Wnt/β and PI3 K, and controls cell proliferation, cell cycle signaling, and DNA repair (Jianing et al. 2021). In addition, GSK3β through induction of inflammation and oxidative stress can induce the development of neurodegenerative diseases such as AD (Llorens-Marítin et al. 2014). Of note, Aβ and hyperphosphorylated tau protein promote the expression of neuronal GSK3β which also induce the activation of APP and the phosphorylation of tau protein (Llorens-Marítin et al. 2014). Takashima (2012) illustrated that mutant PS1 gene induces tau hyperphosphorylation via GSK3β. Activated GSK3β is intricate in AD neuropathology by inducing synaptic dysfunction and the development of cognitive impairment (Llorens-Marítin et al. 2014). However, the exact causes for the up-regulation of GSK3β in AD are not fully elucidated. GSK3β is normally inhibited by insulin and insulin-like growth factor which are downregulated in brain insulin resistance a hallmark of early AD (Zhang et al. 2018). In AD, GSK-3β plays a significant role in hyperphosphorylation of tau protein and is intricate in the insulin/PI3 K/Akt signaling pathway. Dysfunction of the insulin/PI3 K/Akt signaling pathway, which regulates glucose metabolism in the brain, can lead to tau hyperphosphorylation in the brain of AD patents. Moreover, insulin resistance in T2D may cause Aβ deposition which further aggravate the neurotoxicity; then damage the brain and affect the cognitive function. GSK-3β is considered as a common kinase in insulin signaling transduction and tau protein phosphorylation (Zhang et al. 2018). Interestingly, GSK3β can affect other signaling pathways involved in the development and progression of AD. GSK3β cross-talk with PI3 K-AKT-PP2 A pathway, as up-regulation of GSK-3β leads to an increase in the methylation and activity of PP2 Ac through suppression of protein phosphatase methylesterase-1 expression and phosphorylation of leucine carboxyl methyltransferase 1. PP2 A also regulated GSK-3β phosphorylation. Down-regulation of PP2 A enhanced Ser9 phosphorylation of GSK-3β and inhibited its kinase activity. Thus, GSK-3β and PP2 A regulate each other and control tau phosphorylation both directly and indirectly through each other. Reduction of tau phosphorylation by inhibition of GSK-3β may be more than equalized by inhibition of PP2 A through a shift in phosphatase methylesterase-1/leucine carboxyl methyltransferase 1 balance; PP2 A regulates phosphorylation of tau at Ser262/356, a required site for tau pathology (Wang et al. 2015). These findings suggest targeting PP2 A rather than GSK-3β to inhibit tau pathology. It has been observed that the anti-inflammatory tolfenamic acid decreased the expression of hyperphosphorylated tau in the brain by inhibiting GSK-3β activity, decreasing phosphorylated PP2 A, and enhancing PP2 A activity in AD models (Zhang et al. 2020). Moreover, GSK-3β activates PTEN in human neuroblastoma SH-SY5Y cells through induction of oxidative stress. Besides, Aβ results in overexpression of PTEN, a negative regulator of PIP3 (Huang et al. 2014). Curcumin depresses Aβ-induced up-regulation of PTEN induced by Aβ. Thus, curcumin inhibits Aβ-induced tau hyperphosphorylation involving PTEN/Akt/GSK-3β pathway (Huang et al. 2014). Furthermore, inhibition of brain GSK3β by ginsenoside and tolfenamic acid results in the attenuation of AD neuropathology (Yang et al. 2022; Zhang et al. 2020). Indeed, regulating the Wnt/GSK-3β/β-catenin signaling pathway by ginsenoside alleviate oxidative stress, neuro-inflammation, protect neurons, and improve the cognitive impairment in AD (Yang et al. 2022; Zhang et al. 2020) (Fig. 6).

## Targeting of PP2 A/GSK3β/PTEN Axis in AD

Many studies highlighted that PP2 A, GSK3β, and PTEN signaling are interacted mutually in AD development (Kerr et al. 2006; Gürsel et al. 2015). In addition, PTEN overexpression can increase tauopathy by reducing the activity of ERK1/2 independent of PP2 A suppression (Kerr et al. 2006). As well, GSK3β affects the activity of PP2 A during induction of apoptosis (Gürsel et al. 2015). Interestingly, PTEN negatively regulates PI3 K/AKT signaling which has a neuroprotective effect against AD neuropathology by increasing neuronal survival (Matsuda et al. 2018). However, exaggerated GSK3β a downstream of PI3 K/AKT signaling may lead to the induction of neuronal apoptosis. Dysfunction of PI3 K/AKT signaling in AD can induce abnormal activation of GSK3β leading to hyperphosphorylation of tau protein (Matsuda et al. 2018). Qian et al., (Qian et al. 2010) illustrated that PP2 A controls tau protein phosphorylation directly or indirectly via regulating the expression of GSK3β. Moreover, inhibition of PP2 A triggers the activation of GSK3β signaling and increasing the hyperphosphorylation of tau protein (Wang et al. 2015). Importantly, oxidative stress, mitochondrial dysfunction, and neuro-inflammation induce dysregulation of the GSK3β/PTEN/PP2 A axis in AD. In addition, dysregulated GSK3β/PTEN/PP2 A axis trigger oxidative stress, mitochondrial dysfunction, and neuro-inflammation in AD (Kerr et al. 2006; Gürsel et al. 2015).

Interestingly, there are no known upstream regulators or downstream effects of the GSK3β/PTEN/PP2 A axis that could be potential therapeutic targets. Therefore, direct targeting of this axis could be reasonable in treating AD and other neurodegenerative diseases (Matsuda et al. 2018). As well, till now there are no any molecules or drugs selectively affecting the GSK3β/PTEN/PP2 A axis (Matsuda et al. 2018). These findings indicated an interrelated interaction in the PP2 A/GSK3β/PTEN axis in AD. Therefore, inhibition of GSK3β and PTEN and activation of PP2 A may be more suitable than modulation of single signaling pathway.

Furthermore, targeting of PP2 A/GSK3β/PTEN axis may associate with numerous adverse effects. PTEN and/or activating mutations in the proto-typical lipid kinase PI3 K have emerged as some of the most frequent events associated with human cancer and as a result the PI3 K pathway has become a highly sought-after target for cancer therapies (Papa and Pandolfi 2019). GSK3β inhibitors induced severe thigmotaxis after changing the platform's location. This may be primarily an anxiety effect of GSK3β inhibition. One report in a congenitally learned helpless rat model, found a deficiency in GSK3β. Therefore, it is possible that constitutive GSK3β may impact non-cognitive behaviors as well. Again these findings suggest that more work needs to be done to not only understand the normal role of GSK3β in behavior, but to develop approaches that limit hyper-activation without reducing constitutive activity (Hu et al. 2009). The role of PP2 A in regulation of MAPK pathway is complex and other as-yet-unidentified regulatory proteins may be involved. Signaling scaffolding proteins, such as KSR1, are crucial for coordinating spatiotemporal control of the function of kinases, phosphatases and other signaling molecules. The role of PP2 A in both positive and negative regulation of MAPK are likely crucial for fine-tuning and precise control of this pathway. There is also cross-talk with many of these pathways as well as other cell cycle promoting pathways, highlighting the importance of phosphatases in regulating the initiation of the cell cycle (Wlodarchak and Xing 2016).

### Metformin

Metformin is an antidiabetic drug used in the treatment of type 2 diabetes (T2D) (Al-Kuraishy et al. 2023b). Metformin is an insulin-sensitizing drug that inhibits hepatic glucose output through activation of AMP-activated protein kinase (AMPK) (Al‐Kuraishy et al. 2022). In addition, metformin has anti-inflammatory, antioxidant, and anti-apoptotic effects (Al-Kuraishy et al. 2020d; Al-Kuraishy et al. 2023a, 2018). Moreover, metformin may be effective in AD by inhibiting the formation of amyloid plaque and NFTs (Sanati et al. 2022). At molecular level, metformin can affect PP2 A/GSK3β/PTEN axis in AD by different ways (Al-Kuraishy et al. 2020b).

Metformin has a cytoprotective effect against the development of lung cancer by suppressing the expression of GSK3β (Luo et al. 2019). In addition, metformin protects dopaminergic neurons in the substantia nigra by activating AMPK and inhibiting GSK3β (Su et al. 2021). Findings from preclinical study observed that metformin improves cognitive impairment in AD rat model by attenuating the development of brain insulin resistance through inhibition of GSK3β signaling (Kumar et al. 2023). Besides, metformin reduces AD neuropathology by inhibiting tau protein hyperphosphorylation via inhibition of GSK3β signaling (Kumar et al. 2023). Khezri et al., (Sayas and Ávila 2021) proposed according to the preclinical and clinical studies that metformin decrease AD neuropathology by suppressing the expression and activity of GSK3β signaling. GSK3β promotes tau protein hyperphosphorylation and Aβ production and exaggeration of AD neuropathology (Sayas and Ávila 2021). Therefore, metformin by inhibiting GSK3β can reduce the progression of AD.

Moreover, metformin activates the neuroprotective PP2 A thereby may reduce the development and progression of AD (Kickstein et al. 2010). Metformin attenuates tauopathy by activating PP2 A independent of AMPK activation in primary cortical neurons (Kickstein et al. 2010). Pretreatment with metformin prevents microcystin-leucine-arginine-induced neurotoxicity and AD by activating PP2 A and inhibition of GSK3β (Zhang et al. 2021b). Metformin activates PP2 A via inhibition of mTOR signaling in SH-SY5Y (Zhang et al. 2021b). Moreover, metformin through activation of PP2 A inhibits neuro-inflammation by suppressing the expression of NF-κB, NLRP3 inflammasome, and miR-141 in AD mouse model (Docrat et al. 2021). Thus, metformin through activation of PP2 A can reduce AD neuropathology and neuro-inflammation.

Furthermore, metformin through AMPK-dependent pathway regulates vascular inflammation through modulation of PTEN signaling (Kim and Choi 2012). Of note, PTEN signaling negatively regulates of insulin signaling and associated with the development of insulin resistance in T2D (Lee et al. 2011). PTEN negatively regulate brain insulin sensitivity leading to the development of brain insulin resistance (Gupta and Dey 2012). It has been shown that metformin inhibits the expression of *PTEN* gene in preadipocyte 3 T3-L1 cells via AMPK-dependent pathway (Lee et al. 2011). Thus, the insulin-sensitizing effect of metformin is partly mediated by the inhibition of PTEN signaling.

**Fig. 7:**
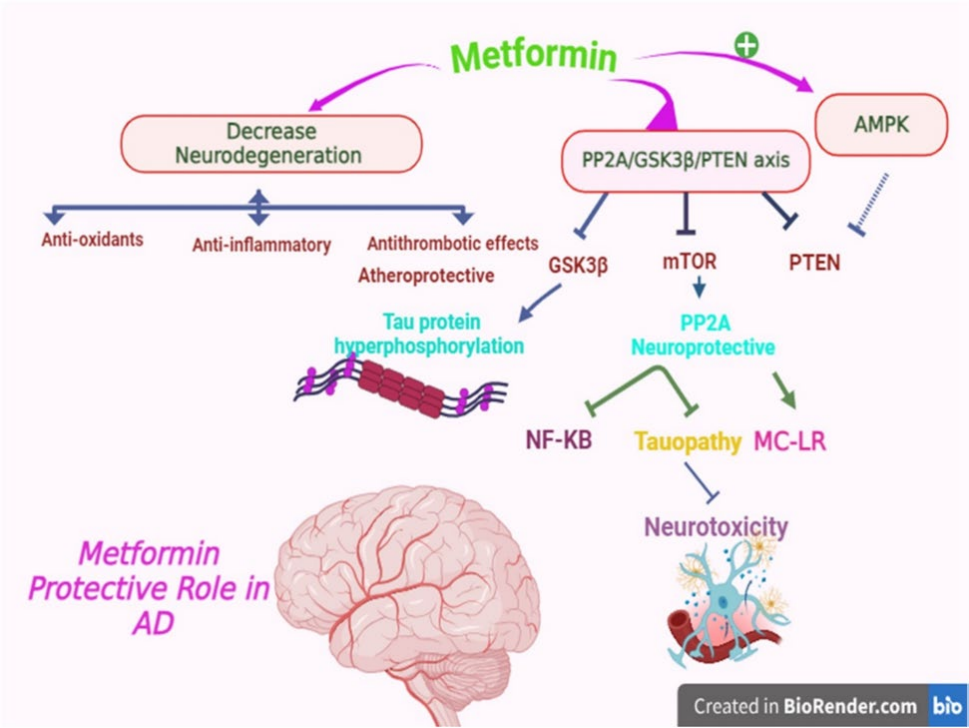
Role metformin in AD: Metformin by activating PP2 A and inhibiting of GSK3β attenuates the development and progression of AD. Metformin by its anti-inflammatory, antioxidant, and anti-apoptotic effects attenuates neurodegeneration in AD. As well, metformin inhibits neurotoxicity by reducing different signaling pathways such as NF-κB and MC-LR

These findings pointed out that metformin by activating PP2 A and inhibiting of GSK3β attenuates the development and progression of AD (Al-Kuraishy et al. 2023e) (Fig. 7).

### Statins

Statins are cholesterol-lowering drugs that widely used in the management of dyslipidemia and cardiovascular disorders (Al-kuraishy et al. 2020a). Statins inhibit de novo *synthesis* of cholesterol by inhibiting liver hydroxyl methyl-gutaryl coenzyme A (HMG-CoA) reductase (Al-Kuraishy and Al-Gareeb 2017). In addition, statins have anti-inflammatory, antioxidant and antithrombotic effects through cholesterol dependent and independent mechanisms (Al-Kuraishy et al. 2021a, b, 2020c; Al-Kuraishy and Al-Gareeb 2016; Kadhim et al. 2019). Furthermore, statins have neuroprotective effects against different neurodegenerative diseases (Al-Kuraishy et al. 2023c, 2024a, b; Alsubaie et al. 2022). However, the effects of statins on the cognitive function and risk of AD is controversial, may be beneficial or detrimental. According to the assorted view of preponderance, the effect of statins seems beneficial against the development of AD. Many observational and epidemiological studies revealed that statins reduced AD risk (Lin et al. 2015; Haag et al. 2009). A systematic review and meta-analysis indicated that statins have beneficial effects against AD development (Xuan et al. 2020). The neuroprotective effects of statins in AD are related to inhibition of Aβ, tau protein hyperphosphorylation, neuro-inflammation and oxidative stress (Sun et al. 2003). As well, statins inhibit brain ApoE4 and HMG-CoA thereby reducing the harmful effect of brain cholesterol in the induction of AD (Leduc et al. 2015).

Furthermore, statins affect PP2 A/GSK3β/PTEN axis in AD by different ways. It has been shown that statins increase the expression of brain PP2 A in ischemic stroke rat model (Zhu et al. 2012). A lipid-soluble simvastatin activates the expression of neuronal PP2 A which inhibited by glutamatergic overactivity in ischemic stroke rat model (Zhu et al. 2012). Li et al., (Li et al. 2015) found that lovastatin inhibits tau protein hyperphosphorylation via activation of neuronal PP2 A in primary cortical neurons. Findings from in vitro study demonstrated that atorvastatin attenuates diabetic cardiomyopathy by activating PP2 A signaling pathway (Ren et al. 2016). Furthermore, atorvastatin downregulates the activity of GSK3β in mice with diabetic cardiomyopathy (Ren et al. 2016). In addition, statins have neuroprotective effects by activating PI3 K/AKT signaling, and inhibition of GSK3β in experimental intracerebral hemorrhage (Yang et al. 2012). Furthermore, atorvastatin has an antidepressant effect in animal model by inhibiting GSK3β signaling pathway (Ludka et al. 2016). Interestingly, the neuroprotective effect of statins against neuronal apoptosis is mediated by inhibiting GSK3β signaling pathway (Hunt et al. 2010). Likewise, atorvastatin prevents tau protein hyperphosphorylation by suppressing GSK3β signaling pathway in APP transgenic mice (Zhou et al. 2016).

These verdicts indicated that statins reduce AD neuropathology by activating PP2 A and inhibiting of GSK3β signaling pathways. However, effects of statins on the PTEN are controversial. It has been shown that lovastatin and other statins upregulate the expression of *PTEN* gene through PPARγ (Teresi et al. 2008). Therefore, high dose of statins may induce the development of insulin resistance in T2D by increasing the expression of *PTEN* gene (Birnbaum et al. 2014). However, atorvastatin attenuates *PTEN* gene expression in experimental micro-embolization (Wang et al. 2016). Therefore, statins are regarded as PTEN modulators; they activate normally expressed PTEN to regulate cell survival. In addition, statins inhibit aberrant and mutated PTEN which involved in the development of neurodegeneration and AD.

### Natural Products

Natural products such as curcumin, berberine, and resveratrol have been reported to be effective against the development and progression of AD. Curcumin was developed as an early diagnostic probe based on its natural fluorescence and high binding affinity to Aβ. Because of its multi-target effects, curcumin has protective and preventive effects against AD. Curcumin has been shown to efficiently preserve the normal structure and function of synapses via multiple signaling pathways: anti-amyloid and metal iron chelating properties, anti-oxidation, and anti-inflammatory activities (Huang et al. 2014; Chen et al. 2018). It has been reported that curcumin treatment prevented injury-induced reductions in PP2 A subunit B levels (Shah et al. 2015). Therefore, curcumin maintains levels of PP2 A subunit B in response to cerebral ischemia, which likely contributes to the neuroprotective function of curcumin in cerebral ischemic injury. Curcumin-treated AD rats reduced the levels of Aβ_40_ and Aβ_42_ in the brain and in the plasma in scopolamine-induced AD rats (Das et al. 2019). Moreover, the levels of GSK3β were considerably decreased in AD rats treated with curcumin (Das et al. 2019). Moreover, curcumin inhibits Aβ-induced tau phosphorylation at Thr231 and Ser396, overexpression of HDAC6, and decrease in phosphorylation of GSK-3β. However, Aβ results in overexpression of PTEN, a negative regulator of PIP3. Curcumin depresses Aβ-induced up-regulation of PTEN induced by Aβ. These results imply that curcumin inhibits Aβ-induced tau hyperphosphorylation involving PTEN/Akt/GSK-3β pathway (Huang et al. 2014).

Berberine is an isoquinoline alkaloid, has several pharmacological activities, including antimicrobial, glucose and cholesterol-lowering, and immunomodulatory properties. It has been suggested that berberine may be beneficial to AD by limiting the accumulation of Aβ and NFTs. Increasing evidence has indicated that berberine reduces risk factors for AD and other neurological diseases by inhibiting oxidative stress and neuro-inflammation (Cai et al. 2016; Babalghith et al. 2022; Al-Kuraishy et al. 2019; Al-Naimi et al. 2019; Hussien et al. 2019b, 2019a). Berberine attenuates tau protein hyperphosphorylation through modulating the activity of Akt/GSK-3β and PP2 A. Moreover, berberine promotes autophagic clearance of tau by enhancing the activity of autophagy via the class III PI3 K/BECLIN-1 pathway in transgenic mice (Chen et al. 2020). Thus, berberine could mitigate cognitive decline by targeting the hyperphosphorylation of tau and the autophagic clearance of tau in AD. In addition, berberine activates AMPK and inhibits mTOR/PTEN signaling of the replication stress-induced cellular senescence (McCubrey et al. 2017).

Resveratrol is polyphenol has antiaging effects, and modulates pathomechanisms of many neurological disorders including AD by modulating different signaling pathways (Ahmed et al. 2017). Originally, it has been established that resveratrol protects against cadmium-induced memory loss and tau protein hyperphosphorylation in rats through activating of AMPK/PI3 K/Akt signaling pathway. Resveratrol significantly activates PI3 K/Akt signaling pathway in rat’s brain by activating PP2 A protein and AMPK/PI3 K/Akt-induced inhibition of GSK3β (Shati and Alfaifi 2019). Furthermore, resveratrol has efficacy in AD patients through suppression of the PTEN signaling pathways (Jin et al. 2023).

Therefore, natural products including curcumin, berberine, and resveratrol have neuroprotective effects against AD neuropathology by modulating PTEN/Akt/GSK-3β pathway (Table 1).

**Table 1:** Neuroprotective effects of natural products on AD neuropathology

Natural Product	Key effects	Targeted pathways/mechanisms
Curcumin	- Preserves synaptic structure and function - Reduces Aβ40 and Aβ42 levels - Inhibits tau hyperphosphorylation	- Anti-amyloid & metal chelation - Antioxidant & anti-inflammatory effects - Modulates PP2 A/Akt/GSK-3β/PTEN pathway
Berberine	- Reduces oxidative stress and neuro-inflammation - Attenuates tau hyperphosphorylation - Enhances autophagic clearance of tau	- Modulates Akt/GSK-3β/PP2 A pathway - Enhances PI3 K/BECLIN-1 autophagy pathway - Activates AMPK and inhibits mTOR/PTEN signaling
Resveratrol	- Protects against memory loss & tau hyperphosphorylation - Suppresses PTEN signaling in AD	- Activates AMPK/PI3 K/Akt pathway - Enhances PP2 A-induced inhibition of GSK3β - Suppresses PTEN signaling

### Others

Biologics and small-molecule drugs display neuroprotective effects in AD. Most of the small compounds used in AD therapy are focused toward important enzymes and pathways that are intricate in the processing of tau or Aβ proteins, including γ-secretase inhibitors such as tarenflurbil, avagacestat, and semagacestat, and β-secretase inhibitors such as lanabecestat, verubecestat, and atabecestat. Similar to other BACE-1 inhibitors, umibecestat has been shown to reduce the burden of Aβ in preclinical animals (Weaver 2023; Thawabteh et al. 2024). The effects of small-molecule drugs on PP2 A/GSK3β/PTEN axis in AD are not fully discussed. Interestingly, small molecules inhibit tau hyperphosphorylation and activating of PP2 A signaling pathway (Wang et al. 2021a). Moreover, small molecules such as ZINC21011059 and ZINC21011066 inhibit GSK3β/PTEN axis in AD (Shukla et al. 2019; Dailah 2022).

Furthermore, non-coding RNAs (ncRNAs), especially microRNAs (miRNAs), and long non-coding RNAs (lncRNAs), are implicated in AD. MiRNAs are conserved small ncRNAs that control gene expression posttranscriptionally, while lncRNAs function in many ways. Recent evidence suggests that miRNAs are aberrantly expressed in AD, and these have been implicated in the regulation of Aβ, tau protein hyper phosphorylation, inflammation, and cell death which are the main pathomechanisms of AD. In addition, regulation of miRNAs varies in blood and cerebral spinal fluid may indicate alterations in AD. Together with brain-specific miRNAs, these miRNAs could be potential AD biomarkers (Tan et al. 2013). Importantly, overexpression of ENST00000440246.1 inhibits cell proliferation and increases Aβ expression in SK-N-SH cells by inhibiting PP2 A. However, PP2 A overexpression reverses the effect of ENST00000440246.1 overexpression in SK-N-SH cells (Gao et al. 2024). Overexpression of NDM29 is observed in AD postmortem cerebral cortex that associated with activation of GSK3β/PTEN axis in AD (Lan et al. 2022). Therefore, ncRNAs and lncRNAs dysregulate PP2 A/GSK3β/PTEN axis in AD lead to disease progression.

Taken together, PP2 A/GSK3β/PTEN axis is dysregulated in AD results in progressive neurodegeneration and exaggeration of AD neuropathology. Modulation of this axis by metformin and statins can reduce AD neuropathology. Importantly, metformin and statins may produce synergistic effects as both drugs modulate PP2 A/GSK3β/PTEN axis in AD.

Therefore, additional studies are recommended in this regard. The present review had several limitations including that most of findings came from preclinical studies which are not completely translated into the clinical settings. In addition, comparing the therapeutic potential of targeting the GSK3β/PTEN/PP2 A axis with other known signaling pathways involved in AD was not discussed. In addition, potential biomarkers to assess the efficacy of targeting the PP2 A/GSK3β/PTEN axis in clinical settings were not evaluated.

However, the present review exciting for future researches regarding the efficacy and safety of the GSK3β/PTEN/PP2 A axis modulators regarding the gap of the current researches (Table 2).

**Table 2:** Effects of small molecules and ncRNAs on PP2 A/GSK3β/PTEN axis in AD

Category	Examples	Mechanisms and effects
Small molecules	- Tarenflurbil - Avagacestat - Semagacestat - Lanabecestat - Verubecestat - Atabecestat - Umibecestat	- Inhibit tau hyperphosphorylation - Activate PP2 A signaling pathway - Inhibit GSK3β/PTEN axis
ncRNAs & lncRNAs	- miRNAs (various) - ENST00000440246.1 - NDM29	- Regulate Aβ and tau hyperphosphorylation - Influence inflammation and cell death - Overexpression of ENST00000440246.1 inhibits PP2 A - NDM29 overexpression activates GSK3β/PTEN axis

## Conclusion

AD is a progressive neurodegenerative disease of the brain due to extracellular accumulation of Aβ and intracellular accumulation of hyperphosphorlyated tau protein which form NFT. Many cellular signaling pathways such as PP2 A, GSK3β, and PTEN are intricate in AD neuropathology. PP2 A which involved in the dephosphorylation of tau protein is deregulated in AD and correlated with cognitive impairment. In addition, mutated and overexpressed PTEN is linked with the development of cognitive impairment by constraining the expression and the activity of PP2 A. Furthermore, dysregulation of GSK3β affects Aβ, tau protein phosphorylation, synaptic plasticity, and other signaling pathways involved in the pathogenesis of AD. These findings indicated a close interaction among GSK3β, PTEN, and PP2 A.

PP2 A, GSK3β, and PTEN signaling are interacted mutually in AD development. PTEN overexpression can increase tauopathy by reducing the activity of ERK1/2 independent of PP2 A suppression. PTEN negatively regulates PI3 K/AKT signaling which has a neuroprotective effect against AD neuropathology by increasing neuronal survival. However, exaggerated GSK3β a downstream of PI3 K/AKT signaling may lead to the induction of neuronal apoptosis. Dysfunction of PI3 K/AKT signaling in AD can induce abnormal activation of GSK3β leading to hyperphosphorylation of tau protein. PP2 A controls tau protein phosphorylation directly or indirectly via regulating the expression of GSK3β. Moreover, inhibition of PP2 A triggers the activation of GSK3β signaling and increasing the hyperphosphorylation of tau protein. These findings indicated an interconnected interaction among PP2 A/GSK3β/PTEN axis in AD. Therefore, inhibition of GSK3β and PTEN, and activation of PP2 A may be more suitable than modulation of single signaling pathway. Metformin and statins by activating PP2 A and inhibiting of GSK3β and PTEN attenuate the development and progression of AD.

Taken together, PP2 A/GSK3β/PTEN axis is dysregulated in AD results in progressive neurodegeneration and exaggeration of AD neuropathology. Modulation of this axis by metformin and statins can reduce AD neuropathology. Consequently, additional preclinical and clinical studies are suggested in this regard.

## Data Availability

No datasets were generated or analysed during the current study.
